# Polyphosphate Kinase 1 Is a Pathogenesis Determinant in Enterohemorrhagic *Escherichia coli* O157:H7

**DOI:** 10.3389/fmicb.2021.762171

**Published:** 2021-10-27

**Authors:** Yanli Du, Xiangyu Wang, Zongli Han, Ying Hua, Kaina Yan, Bao Zhang, Wei Zhao, Chengsong Wan

**Affiliations:** ^1^School of Medical Technology and Nursing, Shenzhen Polytechnic, Shenzhen, China; ^2^Department of Gastroenterology, The First Affiliated Hospital of Shenzhen University, Shenzhen Second People’s Hospital, Shenzhen, China; ^3^Department of Microbiology, School of Public Health, Southern Medical University, Guangzhou, China; ^4^Department of Neurosurgery, Peking University Shenzhen Hospital, Shenzhen, China; ^5^Key Laboratory of Tropical Disease Research of Guangdong Province, Guangzhou, China

**Keywords:** polyphosphate kinase 1 (PPK1), polyphosphate (polyP), enterohemorrhagic *Escherichia coli* O157:H7, pathogenesis, RpoS

## Abstract

The *ppk1* gene encodes polyphosphate kinase (PPK1), which is the major catalytic enzyme that *Escherichia coli* utilizes to synthesize inorganic polyphosphate (polyP). The aim of this study was to explore the role of PPK1 in the pathogenesis of Enterohemorrhagic *E. coli* O157:H7 (EHEC O157:H7). An isogenic in-frame *ppk1* deletion mutant (Δ*ppk1*) and *ppk1* complemented mutant (*Cppk1*) were constructed and characterized in comparison to wild-type (WT) EHEC O157:H7 strain EDL933w by microscope observation and growth curve analysis. Survival rates under heat stress and acid tolerance, both of which the bacteria would face during pathogenesis, were compared among the three strains. LoVo cells and a murine model of intestinal colitis were used as the *in vitro* and *in vivo* models, respectively, to evaluate the effect of PPK1 on adhesion and invasion during the process of pathogenesis. Real-time reverse-transcription PCR of regulatory gene *rpoS*, adhesion gene *eae*, and toxin genes *stx1* and *stx2* was carried out to corroborate the results from the *in vitro* and *in vivo* models. The *ppk1* deletion mutant exhibited disrupted polyP levels, but not morphology and growth characteristics. The survival rate of the Δ*ppk1* strain under stringent environmental conditions was lower as compared with WT and *Cppk1.* The *in vitro* assays showed that deletion of the *ppk1* gene reduced the adhesion, formation of attaching and effacing (A/E) lesions, and invasive ability of EHEC O157:H7. Moreover, the virulence of the Δ*ppk1* in BALB/c mice was weaker as compared with the other two strains. Additionally, mRNA expression of *rpoS*, *eae*, *stx1* and *stx2* were consistent with the *in vitro* and *in vivo* results. In conclusion: EHEC O157:H7 requires PPK1 for both survival under harsh environmental conditions and virulence *in vivo*.

## Introduction

Enterohemorrhagic *Escherichia coli* O157:H7 (EHEC O157:H7) is a gram-negative bacterium that was identified as a human pathogen in 1982 and has continued to be a worldwide threat to public health. EHEC O157:H7 is known to cause hemorrhagic colitis (HC), thrombotic thrombocytopenic purpura (TTP), and hemolytic uremic syndrome (HUS), which results in high mortality (Riley et al., 1983; Perna et al., 2001; Zhao et al., 2013). The main pathogenic mechanisms of EHEC O157:H7 include the production of Shiga toxin (Stx), which is primarily responsible for the renal complications and neurological sequelae of EHEC infections, as well as a type III secretion system (TTSS) that enables tight adherence of bacteria to host epithelial cells by inducing characteristic actin cytoskeletal rearrangements and loss of microvillus structure (A/E lesions) (Nguyen and Sperandio, 2012; Davis et al., 2014; Melton-Celsa, 2014; Stevens and Frankel, 2014; Pacheco et al., 2018; Menge, 2020). In recent years, progress has been made in understanding the function and mechanism of TTSS effector proteins (Wang et al., 2017; Hua et al., 2020), and factors that have overlapping effects on TTSS and Stx, such as sRNA *Esr055*, which is involved in the regulation of the preferential colonization in the large intestine and inhibition of the *stx2* (Han et al., 2017) as well as targeted loci associated with sphingolipid biosynthesis (Pacheco et al., 2018).

Shiga toxin, including Stx1 and Stx2 produced by bacteria including *Shigella dysenteriae* serotype 1 and EHEC O157:H7 strain 933 (Lee et al., 2016), act as primary virulence factors. Each ribosome-inactivating holotoxin possess an AB_5_ molecular configuration consisting of a large monomeric A subunit and small homo-pentameric B subunits (Rangel et al., 2005). Once colonized in intestinal epithelial cells, EHEC induces the delivery of Stx and the production of cytokines and chemokines. Toxins pass through the intestinal mucosa, enter the bloodstream and travel to target organs such as the kidneys and Central Nervous System (CNS). After membrane invasion-mediated endocytosis through the toxin receptor Gb3 on the cell surface, Stxs migrate to the Golgi and endoplasmic reticulum (ER). Stx acts as a multifunctional bacterial protein, promoting ER stress, ribotoxic stress, pro-inflammatory responses, apoptosis, and autophagy in host cells (Lee et al., 2021). In addition to cell death by Stxs, various cells such as neutrophils, induce inflammation in the intestine, which leads to damage.

Adhesion and effacing lesions (A/E lesions) are characterized by intimate bacterial attachment to the surface of intestinal epithelial cells, cytoskeletal rearrangements beneath adherent bacteria, formation of characteristic actin-rich “pedestals” and destruction of proximal microvilli (Stevens and Frankel, 2014).

The EHEC TTSS injects a plethora of effector proteins into host cells that induce alteration or disruption of numerous host cell processes (Pacheco et al., 2018). Tir is the first translocated effector protein inserted into the host cell membrane as a receptor of adhesion intimin. Intimin-Tir interactions are required to cluster Tir, which initiates the process of activating actin assembly and recruiting cytoskeletal proteins, such as clathrin (Hua et al., 2018), at the site of bacterial adherence (Campellone et al., 2004a; Stevens and Frankel, 2014). The main pathway causing actin rearrangement resulting in the formation of actin-rich pedestals eventually in EHEC is the Tir: IRTKS/IRSp53:TccP/EspFu pathway, which triggers Arp2/3-mediated actin polymerization independently of N-WASP (Vingadassalom et al., 2010).

Inorganic polyphosphate (polyP) is abundant in all cells, including bacteria, fungi, parasites, plants, and animals, and plays critical roles (Rao et al., 2009). Polyphosphate kinase 1 (PPK1) reversibly catalases the polymerization of the terminal phosphate of ATP into a polyP chain and is the major catalytic enzyme in *E. coli* that synthesizes polyP. PPK1, encoded by the *ppk1* gene, has been related to stationary phase (SP) survival and pathogenesis in many bacteria species, such as *E. coli, Vibrio cholera, Shigella* and *Salmonella spp.*, via polyP inducing the expression of DNA repair enzymes (Varas et al., 2017) or inducing the expression of *rpoS*, which is the selective *σ^*S*^* or *σ^38^* factor of the RNA polymerase and regulates the expression of survival and other virulence genes in bacteria, such as *E. coli* (Shiba et al., 1997). However, RpoS proteins are not the only regulators of survival and virulence genes during the SP in *E. coli*. A previous study identified a new regulatory gene, *ibeR*, that mediated stress resistance and pathogenesis SP gene expression in *E. coli* K1 RS218, which has a loss-of-function mutation in the *rpoS* gene (Chi et al., 2009). Additionally, Varas et al. (2017) found that the metabolic balance of polyP was necessary for the synthesis of the second messenger (p) ppGpp, which regulated other important cellular and regulatory processes such as the recycling of sigma factors/anti-sigma factors that allowed the bacteria to adapt to a wide range of environmental conditions, including nutritional or stressful conditions (Magnusson et al., 2005; Dalebroux and Swanson, 2012). To investigate the role of PPK1 in the pathogenesis of EHEC O157:H7, an isogenic in-frame *ppk1* deletion mutant of EHEC O157:H7 EDL933w and a *ppk1*-complemented strain, where the *ppk1* open reading frame in the plasmid *pBAD33* was transferred into the *ppk1* deletion mutant, were constructed and characterized. The polyP levels in the *ppk1* deletion mutant (Δ*ppk1*) indicated that the Δ*ppk1* was deficient in polyP synthesis as compared to the wild-type (WT) and *ppk1*-complemented (*Cppk1*) strains. Moreover, the deletion of *ppk1* influenced the survival of EHEC O157:H7 under harsh environmental conditions of 55°C and pH 2.0. PPK1 contributed to the adhesion, formation of A/E lesions, and invasion of LoVo cells *in vitro*. Additionally, deletion of the *ppk1* gene reduced the virulence in BALB/c mice. Moreover, deletion of *ppk1* resulted in decreased expression of *rpoS*, as well as the adhesion gene *eae* and virulence genes *stx1* and *stx2*. Our findings suggest that PPK1 is a determinant in the pathogenesis of EHEC O157:H7.

## Materials and Methods

### Bacterial Strains, Cells, Plasmids and Culture Conditions

Enterohemorrhagic *Escherichia coli* O157:H7 strain EDL933w was obtained from the Chinese Center for Disease Control and Prevention (CDC) (Beijing, China). The nalidixic acid resistant mutant of EHEC O157:H7 strain EDL933w named EHEC O157:H7 EDL933w (Nal^*R*^) was selected as described by published paper by Zhao et al. from our lab (Zhao et al., 2013). Plasmids, pKD46 [containing recombinant enzymes including Gam, Bet, and Exo (Exo is an exonuclease that binds to the ends of double-stranded DNA, degrades the DNA from the 5′ end to the 3′ end, and produces 3′ overhangs. Beta binds to the single-stranded DNA and mediates the complementary single-stranded DNA annealing. Gam protein binds with the RecBCD enzyme to inhibit its activity of degrading foreign DNA Jeong et al., 2013)] and pKD4 plasmid (containing the kanamycin resistance gene) were obtained from the Institute of Microbiology and Epidemiology (Beijing, China). LoVo colonic carcinoma cells and DH5α cells with the plasmid pBAD33 (ATCC, 87402) were purchased from ATCC. T4 DNA ligase and restriction endonucleases were purchased from TaKaRa Biotechnology Co., Ltd. (Dalian, China). Standard laboratory reagents and chemicals, such as DAPI, were purchased from Sigma-Aldrich (St. Louis, MO, United States) unless otherwise mentioned. Primers used in this study were synthesized by Sangon Biotech Co., Ltd. (Shanghai, China). DNA sequencing was performed by Guangzhou IGE Biotechnology, Ltd. (Guangzhou, China).

All bacterial strains in this study were grown in Luria broth (LB) media (10 g SELECT Peptone 140 (Oxiod, England), 5 g SELECT Yeast Extract (Oxiod, England), and 10 g sodium chloride (Beijing, China) dissolving completely in double-distilled water (ddH_2_O) with 5mol/L NaOH for pH = 7.0 and a total volume of 1 L by ddH_2_O, autoclave sterilization by 121°C for 20 min) at 37°C at 200 rpm in a constant-temperature, oscillating shaker with nalidixic acid (50 μg/mL) and appropriate antibiotics (WT: no antibiotics; Δ*ppk1*: 100 μg/mL kanamycin; *Cppk1*: 100 μg/mL kanamycin and 10 μg/mL chloramphenicol) if necessary. LoVo cells were cultured in DMEM (Gibco, Waltham, MA, United States) with 10% fetal bovine serum (FBS, Gibco, Waltham, MA, United States) and 1% penicillin/streptomycin (Gibco, Waltham, MA, United States) in 5% CO_2_ for routine passage prior to infection.

Female BALB/c (4–5 weeks of age) mice were obtained from the Lab Animal Center of Southern Medical University (Guangzhou, China; Certificate: SCXK (Guangdong Province) 2016-0041, No. 44002100010995]. All mice were housed under specific pathogen-free (SPF) conditions according to the regulations of the animal care committee. This study was conducted in accordance with the recommendations of the Southern Medical University Experimental Animal Ethics Committee (Guangzhou, China).

### Construction of an Isogenic In-Frame Deletion Mutant of *ppk1* and *ppk1*-Complemented Strains

A pKD46-mediated λ red homologous recombination system was used to construct EHEC O157:H7 Δ*ppk1*. According to the *ppk1* gene sequence in GenBank (Accession no. NC_002655) and its upstream and downstream DNA sequences, three pairs of primers were designed and synthesized (Table 1). H1-K1 and H2-K2 primers contained homologous arms, of which the 5′ end was homologous with the *ppk1* gene and the 3′ end was homologous with the kanamycin resistant gene. PPK-P1, PPK-P2, N1-F, and N2-R were cross-identifying primers specific for the *ppk1* gene in EHEC O157:H7. Kana-F and Kana-R were internal-identifying primers for the kanamycin resistance gene within the pKD4 plasmid. The kanamycin resistance gene was amplified using primers H1-K1 and H2-K2 with the pKD4 plasmid as a template by using overlap extension PCR. The PCR conditions were as follows: 94°C for 3 min, followed by 35 cycles of 94°C for 30 s, 56°C for 30 s, and 72°C for 90 s, and a final extension of 72°C for 10 min. Subsequently, plasmid pKD46 was transformed into EHEC O157:H7 EDL933w and cultured to an OD_600_ = 0.2–0.3. L-arabinose was then added to a final concentration of 10 mmol/L for 40–60 min to induce the full expression of recombinant enzymes Exo, Bet, and Gam of pKD46. Finally, 10 μL of the targeting fragments (34.67 ng/μL) isolated from the gels and the prepared 100 μL EHEC O157:H7 EDL933w competent cells were mixed in a gene pulser cuvette (Bio-Rad Laboratories, Inc., Hercules, CA, United States) and subjected to an electric shock for 10–20 s (25 μF, 200 Ω, 3 KV). Positive strains were screened using LB plates containing 50 μg/mL kanamycin. The recombinant strain was confirmed by PCR and DNA sequencing.

**TABLE 1:** Primer sequences used in the construction of the isogenic in-frame deletion mutant of *ppk1* and *ppk1*-complemented strains.

Primers	Sequences (5′-3′)
H1-K1	5′-GCCATAATATCCAGGCAGTGTC CCGTGAATAAAACGGAGTAAAAGT GGTAGTGTAGGCTGGAGCTGCTTC-3′
H2-K2	5′-CCGCAGCAAACTCCTGCGGACGAGGGGATTTATCG TGTATTGGCATAGGGCATATGAATATCCTCCTTAG-3′
PPK-P1	5′-CGAAGAACAAGGCTCCAAC-3′
PPK-P2	5′-AAGGCAGTAACGCAGAATG-3′
Kana-F	5′-CGGTGCCCTGAATGAACTGC-3′
Kana-R	5′-CGGCCACAGTCGATGAATCC-3′
N1-F	5′-TTTGCCGATGGTCGTCTGA-3′
N2-R	5′-TCCAGCCCTGAATACGAAA-3′
PPK1-F	5′-GATTCTAGA***AGGAGG***AAGT GGTAATGGGTCAGGAAAAGCTATACA-3′
PPK1-R	5′-GACAAGCTTTTATTCAGGTTGTTCGAGTGATT-3′
pBAD-F pBAD-R	5′-ATGCCATAGCATTTTTATCC-3′ 5′-GATTTAATCTGTATCAGG-3′
PK1-F	5′-AATGCGCTGGTTGAAGTGTT-3′
P**K**1-R	5′-CAGCACATGCTCAAAGGTGT-3′
16S rRNA-F	5′-AAGCTGGAATCGCTAGTAATC-3′
16s rRNA-R	5′-TGTGTACAAGGCTCGATGAC-3′

*The primer of PPK1-F: TCTAGA is the restriction site of *Xba* I. ***AGGAGG*** is the Shine-Dalgarno sequence (SD), the synthetic ribosomal binding site. The primer of PPK1-R: AAGCTT is the restriction site of *Hind* III.*

The complemented strain, *Cppk1*, was constructed using the prokaryotic expression plasmid *pBAD33*. A DNA fragment carrying the complete *ppk1* open reading frame was amplified using the PPK1-F and PPK1-R primers (Table 1) with the EDL933w genome as the DNA template. After double-enzyme digestion with *HindIII* and *XbaI*, the DNA fragment and vector *pBAD33* were ligated together with T4 DNA Ligase, and the products were transformed into strain EHEC O157:H7 Δ*ppk1*. Positive strains were screened using LB medium containing 100 μg/mL kanamycin and 10 μg/mL chloramphenicol. PCR and DNA sequencing analyses were conducted to identify the complemented strains. Finally, quantitative real-time PCR using Applied Biosystems (ABI) 7500 FAST Real-Time PCR system (Thermo Fisher, Waltham, MA United States) was performed to verify the transcription of *ppk1* in the complemented strain. Briefly, the WT, Δ*ppk1* and *Cppk1* strains were cultured in liquid LB medium overnight. Total RNA extracted with TRIzol^®^ (Invitrogen, Carlsbad, CA, United States) was reverse transcribed using the ExScript RT reagent kit (Takara, Japan) according to the manufacturer’s instructions. The real-time PCR conditions were: 95°C for 2 min, followed by 40 cycles of 95°C for 15 s and 60°C for 32 s. A 16S rRNA gene fragment (68 bp) was used as an internal control, and the amplification of *ppk1* (255 bp) in the WT strain was used as a reference. Gene expression levels were calculated using the **2^–ΔΔ^*^*C*^*^*T*^** method (Livak and Schmittgen, 2001).

### Gram Staining and Growth Curves

Gram staining was performed with the classical steps (Coico, 2005), and reagents needed for this assay were from Nanjing Jiancheng Technology Co., Ltd., China. The growth curves were determined by measuring the optical density at 600 nm (OD_600_) of the cultures at different times in LB media at 37°C at 200 rpm in a constant-temperature, oscillating shaker. Antibiotics were added if the strain required them as described above.

### Measurement of polyP Levels

Quantification of polyP by DAPI was performed in different fields of view as previously published (Aschar-Sobbi et al., 2008; Diaz and Ingall, 2010; Kulakova et al., 2011; Gomes et al., 2013). Based on these researches, we optimized the procedure in *E. coli*. Briefly, the WT, Δ*ppk1* and *Cppk1* strains were grown in LB media for 12-14 h at 37°C with the appropriate antibiotics. The cells were then diluted to an OD_600_ = 1.0, which equaled approximately 1 × 10^8^ CFU/mL, and centrifuged at 10,000 × *g* for 15 min at 4°C. After washing with 20 mM HEPES buffer (pH 7.5) twice, the pellets were resuspended in 1 mL of 20 mM HEPES buffer (pH 7.5). In order to release intracellular polyP from bound state for direct quantification without prior extraction, cells were lysed by a freeze-thaw cycle at − 80°C followed by thawing at 24–26°C. Then, 300 μL of the lysed cells were added to new sterilized microcentrifuge tubes containing 600 μL of 20 mM HEPES buffer (pH 7.5). Next, 100 μL of 100 μM DAPI reagent was added, resulting in a final DAPI concentration of 10 μM. The reaction was allowed to incubate in the dark for 15 min to ensure steady fluorescence. Then 200 μL of each reaction was added to a 96-well plate in triplicate. A polyP standard calibration curve using sodium phosphate glass type 45 (short for polyP45) was constructed on the same 96-well plate. PolyP45 dilutions were prepared in 20 mM HEPES buffer (pH 7.5) for a polyP relative unit (ru) of 0–1 ru, and the concentration of polyP ranged from 0–3 μM Pi. The polyP45 dilutions were mixed with 100 μM DAPI by vortexing for 7.5 min, incubated for 15 min at 24–26°C in the dark, vortexed again, and then 200 μL of each mixture was added to the 96-well plate in triplicate. The fluorescence module of a microplate reader (Infinite M200 Pro, Tecan, Switzerland) was used to measure the fluorescence value in the dark. The excitation wavelength was 415 nm, and the emission wavelength was 550 nm. The polyP quantity of the three strains was extrapolated from the standard curve. For bacteria cultured *in vitro*, we used the polyP concentration in μM Pi/mL, which translates to the amount of polyP in 1 mL of bacteria at an OD600 = 1.0.

### Survival Rates Under Different Stringent Environmental Conditions

The WT, Δ*ppk1* and *Cppk1* strains bacteria were grown in LB medium for 12-14 h at 37°C. The bacterial culture was diluted in LB medium without antibiotics until the OD_600_ was 1.0 (approximately 1 × 10^8^ CFU/mL). For heat stress, the three strains (prepared as stated above) were incubated at 55°C for 0, 1, 2, 3, 4, 5, 8, 10, and 15 min. Samples collected at these points were serially diluted in LB liquid medium until 10^–6^ and then plated on LB agar plates and cultured for at least for 12 h. The survival rate after heat stress for each culture was calculated by dividing the number of colonies counted after exposure to heat stress by the viable cell number before exposure to stress colonies. For acid tolerance test, the samples (prepared as stated above) were collected by centrifugation. Then they were resuspended in acidified LB (pH 2.0) and incubated at 37°C. Samples were collected at 0, 5, 10, 20, 30, 60, and 120 min. Samples collected at these points were serially diluted in LB liquid medium until 10^–6^ and then plated on LB agar plates and cultured for at least for 12 h. The survival rate after acid tolerance for each culture was calculated by dividing the number of colonies counted after exposure to heat stress by the viable cell number before exposure to stress colonies (Peng et al., 2012).

### Preparation of Bacterial Solution for *in vitro* Adhesion and Invasion Assays

The WT, Δ*ppk1* and *Cppk1* strains were grown overnight in LB medium at 37°C. Then the bacteria solution was diluted with LB medium with no antibiotics until the OD_600_ was 1.0 (approximately 1 × 10^8^ CFU/mL). The bacteria were collected by centrifugation and resuspended in cell culture medium without antibiotics.

### Adherence Quantitation Assay

The adherence assay was carried out according to previous methods (Elsinghorst and Kopecko, 1992; Peng et al., 2012). Briefly, LoVo confluent monolayers in 12-well plates (approximately 1 × 10^6^ cells/well) were incubated with the WT, Δ*ppk1* and *Cppk1* strains of EHEC O157:H7 (1 × 10^8^ CFU/well) for 2 h in an incubator with 5% CO_2_**,** 95% air [including 78% nitrogen, 21% oxygen, 0.934% rare gases (helium, neon, argon, krypton, xenon, and radon)], 37°C. After incubation, the monolayers were washed at least five times with phosphate buffered saline (PBS). Then the cells were lysed with 0.5% Triton X-100 for 10 min, which had no lethal effect on the bacteria. The lysate was then serially diluted at 1:1000 in PBS, plated on LB plates, and incubated for 12 h. The number of colonies was counted, and the adhesion rate was calculated as follows: ‰ = (number of colonies × 10^3^)/1 × 10^8^. Each sample was assayed in triplicate.

### Adherence Assay by Scanning Electron Microscopy

Nine sterile cell slides for 24-well plates were placed in 9 wells of a 24-well plate. Then 200 μl of LoVo cells (approximately 5 × 10^5^/mL) were added into the 9 wells for cultivation in a 5% CO_2_ incubator at 37°C until the cells formed confluent monolayers (approximately 1.0 × 10^6^ cells/well). The WT, Δ*ppk1* and *Cppk1* strains of EHEC O157:H7 with OD_600_ = 1.0 (approximately 1 × 10^8^ CFU/mL of bacteria) were inoculated to those wells at a 1:100 cell:bacteria ratio. The 24-well plate was placed in a 5% CO_2_ incubator at 37°C. After washing with PBS five times, the nine wells were placed into wide caliber EP tubers and fixed with 2.5% glutaraldehyde for SEM.

### Invasion Assay

LoVo confluent monolayers in 12-well plates (approximately 1 × 10^6^ cells/well) were incubated with bacteria (1 × 10^8^ CFU/well) for 2 h. The monolayers were washed with PBS and incubated with the mixed medium [DMEM with 10% heat-inactivated FBS and gentamicin (100 μg/mL)] for another 2 h at 37°C in order to kill the extracellular bacteria. The monolayers were then washed with PBS and lysed with 0.5% Triton X-100. The released intracellular bacteria were collected, serially diluted, plated on LB plates and cultured for 12 h. The number of bacteria was counted, and the invasion rate is expressed as follows: ‰ = (number of bacteria × 1000)/number of total added bacteria. The assay was performed in triplicate for each strain.

### Invasion Assay via Transmission Electron Microscopy

All the procedures were the same as described above (i.e., “Invasion assay” section). After treatment with gentamicin for 2 h at 37°C, the monolayers were washed with warm cell culture medium. The cells were then scraped off the plates and fixed with 2.5% glutaraldehyde for transmission electron microscopy (TEM) imaging.

### Intracellular F-Actin Observation in LoVo Cells Infected by Enterohemorrhagic *Escherichia coli* O157:H7 via Confocal Microscopy

LoVo cells were passaged in confocal culture dishes (35 mm in diameter) and cultured into monolayers. The bacterial culture and invasion processes are described above (i.e., “Invasion assay” section). The culture media was removed, and the cells were gently washed once with phosphate buffered saline (PBS) at 37°C. Then cells were fixed in 4% paraformaldehyde for 10 min at 24–26°C, washed with PBS for 30 s, and then permeabilized with 0.15% Triton X-100 for 5 min. The cells were blocked with 1% bovine serum albumin after washing with PBS at 24–26°C for 5 min, and then 100 nM rhodamine phalloidin was added and incubated with the cells for 30 min in the dark at 37°C. After washing three times in PBS, the DNA was counterstained with 100 nM DAPI in PBS for 30 s. The samples were rinsed with PBS, and then confocal images (LSM710, ZEISS, Germany) of intracellular actin were obtained.

### Murine Model of Infectious Enterohemorrhagic *Escherichia coli* O157:H7 Colitis

The animal experiments were performed strictly according to the guidelines for animal care at Southern Medical University (Guangzhou, China). The murine model of *E. coli*-induced colitis was performed as described previously (Zhao et al., 2013). In brief, twenty BALB/c mice (4–5 weeks old) were randomly divided into four groups: (1) EHEC WT, (2) Δ*ppk1*, (3) *Cppk1*, and (4) control (uninfected). The mice were provided water containing nalidixic acid (50 μg/mL) for 12 h prior to infection. Mice were then infected twice at an interval of 12 h. Mice were simultaneously administered 300 μL of the bacterial suspension (2 × 10^10^ CFU/mL total) orally and then intraperitoneally injected with mitomycin C (MMC, 2.5 mg/kg) to improve their infection susceptibility. Mice in the control group were treated with MMC and inoculated equivalently with LB broth under the same conditions. The mice were sacrificed at day 7 post-infection. The distal 5 cm of the colon was harvested. Typical features of the colon, such as the appearance of the colons and solidified feces, were compared among the groups. After cleaning with PBS buffer, the colons were embedded in paraffin and stained with hematoxylin and eosin (H&E) to observe the pathological changes among the groups. Histologic damage scores were performed using previously described methods (Ledwaba et al., 2020) by a pathologist who had no knowledge of this study.

### Real-Time Reverse-Transcription PCR

The mRNA expression levels of *rpoS* (encodes RpoS), *eae* (encodes intimin), *stx1A*, *stx1B* (both encode Stx1), *stx2A* and *stx2B* (both encode Stx2) in WT, Δ*ppk1*, and *Cppk1* strains were determined by quantitative real-time PCR. Primers (Table 2) were synthesized and used for cDNA amplification of 16S rRNA (68 bp), *rpoS* (225 bp), *eae* (220 bp), *stx1A* (240 bp), *stx1B* (112 bp), *stx2A* (129 bp), and *stx2B* (111 bp). A 16S rRNA gene was used as an internal control. All gene expression was normalized against WT of EHEC O157:H7 using the **2^–ΔΔ^*^*CT*^*** method (Livak and Schmittgen, 2001).

**TABLE 2:** Primers used in qPCR.

Primers	Sequences (5′-3′)
*rpoS*-F	5′-CAGCTTATGGGACAACTCAC-3′
*rpoS*-R	5′-GCGTTGCTGGACCTTATC-3′
*eae*-F	5′-GCATTAAGTGCTGAAGTCAT-3′
*eae*-R	5′-ACGCCGATACCATTACTTAT-3′
*stx1A*-F	5′-GCAGGACACTACTCAACCTT-3′
*stx1A*-R	5′-ATCGCCATTCGTTGACTACT-3′
*stx1B*-F	5′-AAGCTTCAGCTGTCACAGTA-3′
*stx1B*-R	5′-CGCCATTCGTTGACTACTTC-3′
*stx2A*-F	5′-TCTGGCGTTAATGGAGTTCA-3′
*stx2A*-R	5′-AGTGCCTGACGAAATTCTCT-3′
*stx2B*-F	5′-TGACGGGAAAGAATACTGGA-3′
*stx2B*-R	5′-GAGCCTGATTCACAGGTACT-3′
16S rRNA-F	5′-AAGCTGGAATCGCTAGTAATC-3′
16S rRNA-R	5′-TGTGTACAAGGCTCGATGAC-3′

### Statistical Analysis

Experimental values are expressed as the mean ± standard deviation. A Student’s *t*-test was used to analyze values between two groups, while a one-way ANOVA was used to analyze values between three groups. Multiple comparisons were completed by using the Student-Newman-Keuls (S-N-K) and Bonferroni tests. Differences with *p* < 0.05 were regarded as statistically significant.

## Results

### Construction of Enterohemorrhagic *Escherichia coli* O157:H7 Δ*ppk1* and *Cppk1* Strains

**FIGURE 1:**
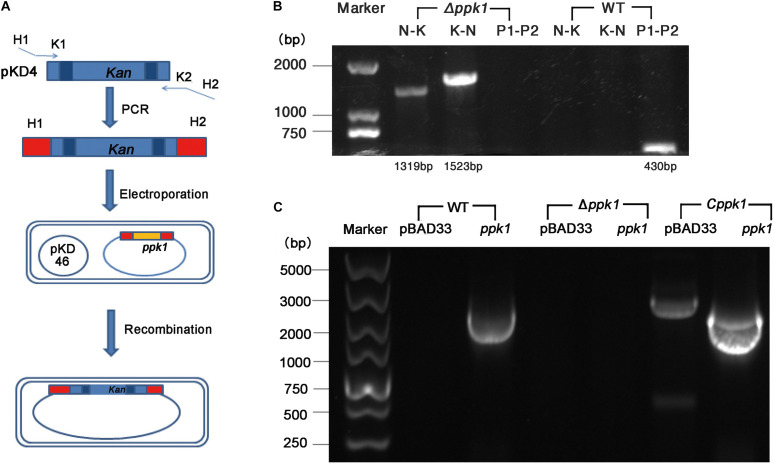
Construction of the *ppk1* deletion mutant (Δ*ppk1*) and complemented strain (*Cppk1*) of EHEC O157:H7. **(A)** Schematic diagram of the λ red system homologous recombination. **(B)** The primers of lane 2 and 5 (N-K) were N1-F and Kana-R. The primers of lane 3 and 6 (K-N) were Kana-F and N2-R. The primers of lane 4 and 7 (P1-P2) were PPK-P1 and PPK-P2. **(C)** Two pairs of primers (*pBAD33-F/R* and *ppk-F/R*) were used to verify the complemented strain, which produced products of 2092 bp and 2268 bp, respectively. The *Cppk1* strain contained both fragments, while WT strain contained only the 2092 bp, and the Δ*ppk1* strain contained neither.

The *ppk1* isogenic in-frame deletion mutant strain of EHEC O157:H7 EDL933w was successfully constructed. As shown in Figure 1A, *ppk1* was replaced by a kanamycin resistance cassette, both of which contained the same homologous arm H1 and H2. Deletion of the *ppk1* gene was initially confirmed by colony PCR (Figure 1B). All three pairs of primers verified the successful deletion of *ppk1* from the Δ*ppk1* strain, and DNA sequencing further confirmed this deletion.

Similarly, the *Cppk1* strain was constructed based on the Δ*ppk1* and verified by colony PCR (Figure 1C) and DNA sequencing. The results of real-time reverse-transcription PCR displayed that the *ppk1* gene in the Δ*ppk1* was minimally expressed as compared with WT (*t* = 18.227, *P* < 0.001), which indicated that the *ppk1* gene was successfully knocked out in Δ*ppk1*. The *ppk1* gene was increased 58-fold in the *Cppk1* strain as compared to WT (*t* = −16.869, *P* < 0.001), demonstrating that 0.2% L-arabinose could induce a high expression of recombinant plasmid in the *Cppk1* strain.

### The Influence of the *ppk1* Deletion Mutant in polyP Levels, Morphology and Growth Characteristics

**FIGURE 2:**
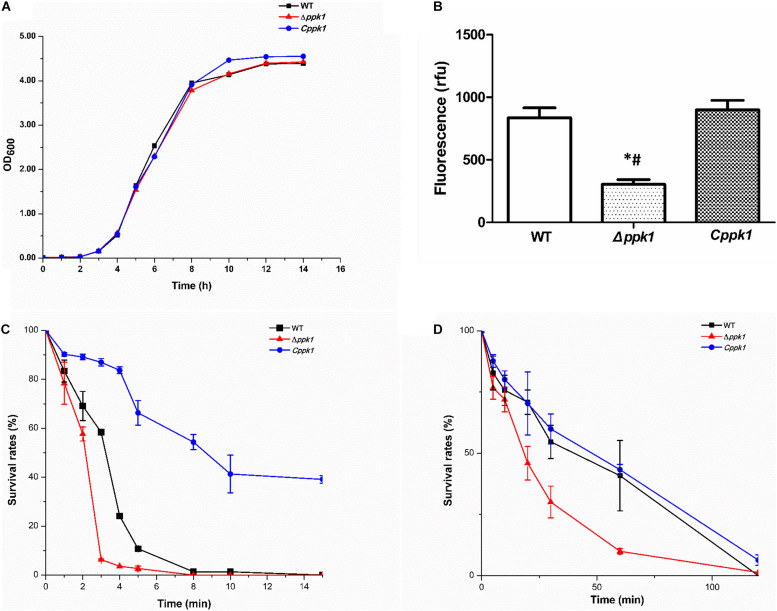
Comparison of the biological characteristics and stringent stress survival assays of the three EHEC O157:H7 strains. **(A)** Growth curve analysis of the WT, Δ*ppk1*, and *Cppk1* strains. OD: Optical density. **(B)** The polyP levels of WT, Δ*ppk1*, and *Cppk1* strains. Error bars indicate standard deviations. *, *p* < 0.05 as compared with the WT strain; #, *p* < 0.05 as compared with the *Cppk1* strain. **(C)** The survival rates of WT, Δ*ppk1*, and *Cppk1* strains after exposure to 55°C at different time points. **(D)** The survival rates of WT, Δ*ppk1*, and *Cppk1* strains under acid stress at pH 2.0 in liquid LB at different time points.

The growth curve of the EHEC O157:H7 strain Δ*ppk1* displayed an identical trend of growth as that of the WT and *Cppk1* strains in LB medium (Figure 2A). All three strains entered the exponential phase at 3 h post-inoculation and stationary phase at 12 h post-inoculation. In addition, there were no significant differences in the morphology of the bacteria as revealed by optical microscopy and scanning electron microscopy (data not shown). Yet, based on the results Δ*ppk1* was defective in polyP synthesis. DAPI staining was used to quantify the polyP levels of the three strains. The results demonstrated that polyP levels were significantly different (*F* = 38.90, *P* < 0.001) among the strains. The fluorescence of the Δ*ppk1* strain [304.19 relative fluorescence units (rfu)] was significantly lower than that in the WT (835.38 rfu) and *Cppk1* (899.36 rfu) strains (Figure 2B), with no significant differences between WT and *Cppk1*. This indicated that complementation of the Δ*ppk1* with the *ppk1* gene enabled the *Cppk1* strain to produce polyP. All factors above indicated that the deletion of *ppk1* did not change the morphology and growth rate of the Δ*ppk1* in nutritive medium but significantly reduced the polyP level.

### Deletion of the *ppk1* Gene Affected the Ability of Enterohemorrhagic *Escherichia coli* O157:H7 to Survive Under Stringent Environmental Conditions

When the Δ*ppk1* strain was incubated at 55°C (EHEC O157:H7 can withstand this temperature) for 3 min, the survival rate was only 6.31% as compared with 58.39% for the WT strain. *Cppk1* (86.96%) was more tolerant to heat than that of WT and Δ*ppk1* strains (Figure 2C). When the bacterial strains were suspended and cultured in liquid LB at pH 2 (close to the acidic environment of the human stomach) for 60 min, similar results to the heat shock experiment were obtained. The survival rate of the Δ*ppk1* was only 9.97% as compared with 40.84% of the WT and 43.27% of *Cppk1* (Figure 2D). Both the heat shock and strong acid resistance results suggested that *ppk1* played an important role in the stringent response in EHEC O157:H7.

### Deletion of the *ppk1* Gene Changed the Adhesion Capacity to Cells, F-Actin Rearrangement in Cells and Adhesion and Effacing Lesion Formation of Enterohemorrhagic *Escherichia coli* O157:H7

**FIGURE 3:**
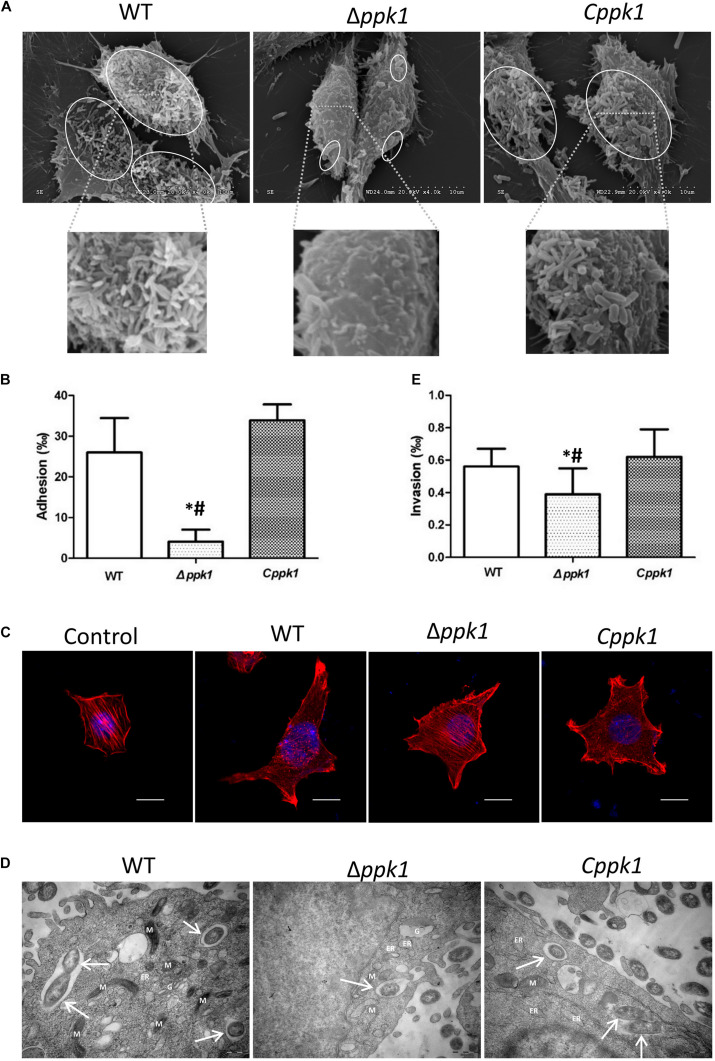
Deletion of the *ppk1* gene reduced the adhesion, A/E lesions, and invasive ability. **(A)** High-resolution SEM images of LoVo cells infected with WT, Δ*ppk1*, or *Cppk1* strains. Scale bar: 10 μm. **(B)** Comparison of adhesion rates between the WT, Δ*ppk*1, or *Cppk*1 strains. **(C)** LoVo cells were stained with rhodamine phalloidin (actin) and DAPI (nuclei) after infection. Scale bar: 10 μm. **(D)** High-resolution TEM images of LoVo cells infected with WT, Δ*ppk1*, or *Cppk1* strains. White arrows point to the bacteria. ER, endoplasmic reticulum; M, mitochondria; G, Golgi apparatus. Scale bar: 500 nm. **(E)** Comparison of invasion rates between the WT, Δ*ppk*1, and *Cppk*1 strains. Error bars in both **(B)** and **(E)** indicate standard deviations. *, *p* < 0.05 as compared with the WT strain; #, *p* < 0.05 as compared with the *Cppk*1 strain. **(E).**

Scanning electron microscopy (SEM) results of the adhesion assay demonstrated that the three bacterial strains adhered to LoVo cells, but Δ*ppk1* accumulated on and adhered to LoVo cells at a lower amount as compared to the WT and *Cppk1* strains (Figure 3A) under each magnification. These results were verified through an adhesion quantitative assay (*F* = 24.680, *P* < 0.001). The adhesion rate of the Δ*ppk1* was only 4.09‰ as compared with WT (26.02‰) and *Cppk1* (33.88‰) strains (Figure 3B).

F-actin was stained with rhodamine phalloidin and observed by confocal microscopy. Results showed that the actin filaments of LoVo cells in the control group were typically present, distributed evenly, and arranged neatly in the cells (Figure 3C), whereas the F-actin in the WT and *Cppk1* groups were significantly reduced and rearranged with significant aggregating at the two ends of cells. When the *ppk1* gene was knocked out, the ability to induce actin rearrangement was significantly weaker than that of WT and *Cppk1* strains. These results demonstrated that PPK1 plays an important role in EHEC O157:H7 in the development of A/E lesions.

### Deletion of the *ppk1* Gene Affected the Invasive Ability of Enterohemorrhagic *Escherichia coli* O157:H7

Transmission electron microscopy results demonstrated that the three strains all invaded LoVo cells and were wrapped in vacuoles (Figure 3D). However, the Δ*ppk1* was less invasive as compared to the WT and *Cppk1* strains. We thus performed gentamicin protection experiments to quantify the invasion rates. The WT reached an invasion rate of 0.56‰, which was not significantly different than the *Cppk1* strain (0.62‰). However, the invasion rate of the Δ*ppk1* was 0.39‰, which confirmed that the *ppk1* gene was also required for bacteria invasion (*F* = 5.224, *P* = 0.014; Figure 3E).

### The Δ*ppk1* Strain Was Less Pathogenic in BALB/c Mice

Twenty BALB/c mice were divided into four groups and infected with EHEC O157:H7 WT, Δ*ppk1, Cppk1*, or LB (control). Mice infected with the WT strain for 2 days showed some symptoms of illness, such as listlessness, anorexia, slow movements, and diarrhea. These symptoms, including a disheveled coat, became more severe the days following the infection until euthanasia on day 7 post-infection. The symptoms of mice in *the Cppk1* group were similar to the WT group, with one mouse dying 5 days post-infection and others exhibiting bloody diarrhea. Mice infected with the Δ*ppk1*, however, exhibited listlessness, rage, and slow movements without obvious diarrhea 3 days post-infection. All mice began to recover gradually in the following days. The uninfected control group treated only with LB were healthy.

**FIGURE 4:**
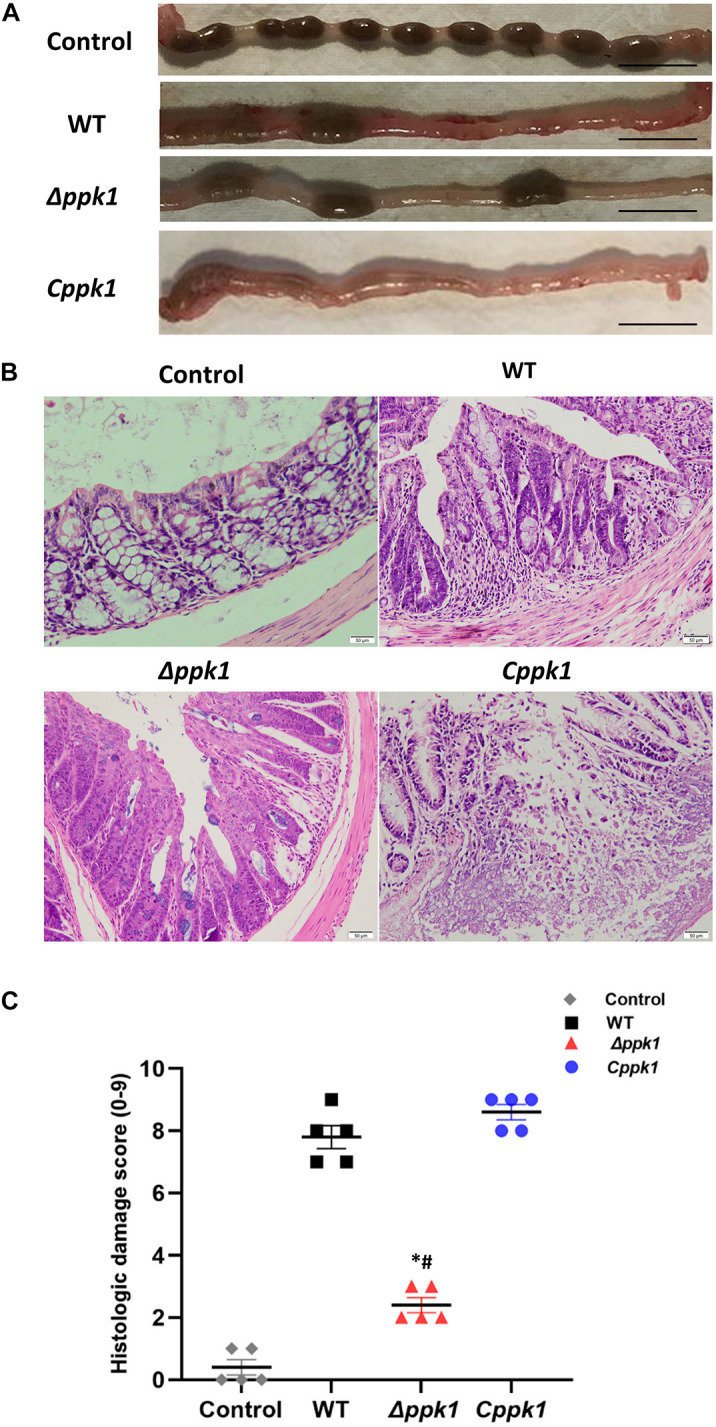
The Δ*ppk*1 strain was less pathogenic in BALB/c mice. **(A)** The colonic morphology of BALB/c mice after the infection. Scale bar: 1 cm. **(B)** Images of colons after infection. Scale bar: 50 μm. **(C)** Histologic damage scores of uninfected (control group), WT, Δ*ppk*1, and *Cppk*1 strains. *, *p* < 0.05 as compared with the WT strain; #, *p* < 0.05 as compared with the *Cppk*1 strain.

After 7 days of infection, the distal 5-cm of the colon was collected, and the pathological features were compared among the groups (Figure 4A). The colon of the control group was normal with solidified stool (Figure 4A), while the WT group displayed edematous and congested intestinal colitis with very minimal solidified stool (Figure 4A). The colon of the mice infected with the *Cppk1* strain exhibited typical intestinal colitis with severe edema and congestion, without any solidified feces (Figure 4A). However, the colon of the mice infected with the Δ*ppk1* group appeared healthier with a small amount of solid stool, although some portions appeared slightly swollen (Figure 4A).

Colon paraffin sections were created and stained with H&E to observe the architecture of the intestinal epithelium (Figure 4B). In the control group, the colonic epithelium was complete and continuous, with regular, clear, and structural glands without inflammatory cell infiltration. Infection with WT of EHEC resulted in mucosal reactionary hyperplasia, disordered cell arrangement, decreased goblet cells, and inflammatory cell infiltration. The *Cppk1* strain caused mucosal necrosis, and the muscle layer was involved and destructed. The colonic mucosa in mice infected with the Δ*ppk1* strain appeared to have reactive hyperplasia, but the glands were regular with a clear structure. The histopathological scores were consistent with the morphological results observed by H&E staining (Figure 4C). The above results demonstrated that the Δ*ppk1* was less pathogenic and induced milder A/E lesions than the WT and *Cppk1* strains. The mild microvilli effacement in the Δ*ppk1* indicated an important role of PPK1 in colonization of the intestinal epithelium.

### *ppk1* Was Required in Modulating the Expression of *rpoS*, *eae, stx1*, and *stx2*

**FIGURE 5:**
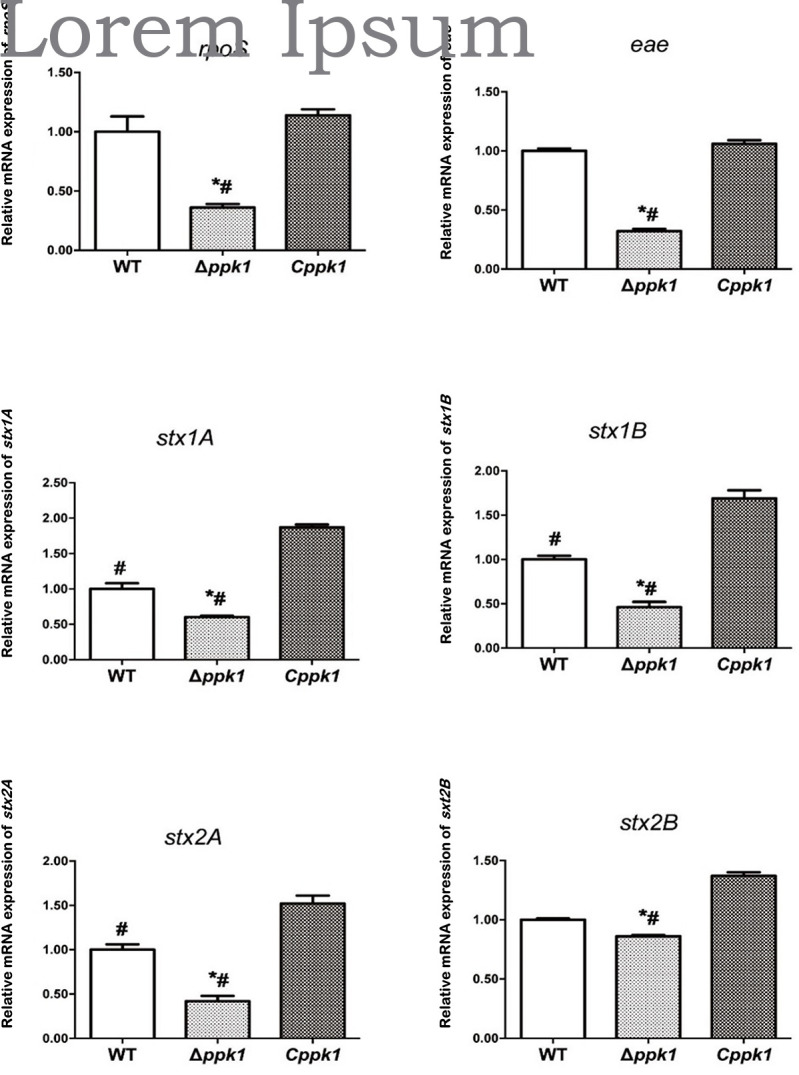
The *ppk1* gene regulated the mRNA expression of *rpoS*, *eae*, *stx1A*, *stx1B*, *stx2A*, and *stx2B*. The relative mRNA expression of *rpoS*, *eae*, *stx1A*, *stx1B*, *stx2A*, and *stx2B* of WT, *ppk1* and *Cppk1* were measured via real-time PCR. Data were normalized to the relative RNA expression of the 16S rRNA. *, *p* < 0.05 as compared with the WT strain; #, *p* < 0.05 as compared with the *Cppk*1 strain.

To determine whether *ppk1* regulated the expression of regulatory gene *rpoS*, adhesion gene *eae*, and virulence genes *stx1* and *stx2*, real-time PCR was conducted. A 16S rRNA gene was used as the internal reference, and WT EHEC was used as the control. The relative level of mRNA in the Δ*ppk1* and *Cppk1* strains was also calculated (Figure 5). The relative copies of *rpoS* in the Δ*ppk1* (0.36 ± 0.03) were less than that in the WT strain (1.00 ± 0.13), while there were no differences between *Cppk1* (1.14 ± 0.05) and WT. These results were consistent with the amount of polyP among the three strains. Moreover, the *ppk1* deletion significantly reduced the relative copy numbers of *eae* (0.32 ± 0.02), *stx1A* (0.60 ± 0.02), *stx1B* (0.46 ± 0.06), *stx2A* (0.42 ± 0.02), and *stx2B* (0.86 ± 0.01) as compared to that of WT (1.00 ± 0.02, 1.00 ± 0.08, 1.00 ± 0.04, 1.00 ± 0.06 and 1.00 ± 0.01, respectively). Similarly, these relative expression levels increased to 1.06 ± 0.03, 1.87 ± 0.04, 1.69 ± 0.09, 1.53 ± 0.01, and 1.36 ± 0.03, respectively, in the *Cppk1* strain as compared with WT. These results indicated that *ppk1* plays an important role in regulating the expression of *rpoS*, *eae*, *stx1*, and *stx*2.

## Discussion

Polyphosphate is available in living cells and plays an important role in the survival, stress response, and pathogenicity of prokaryotic cells (Rao et al., 2009), which is regulated mainly through the regulation of *rpoS*, a key transcriptional factor. PPK1, encoded by the *ppk1* gene, catalyzes the synthesis of polyP in *E. coli*. At present, the role of PPK1 in the pathogenesis of EHEC O157:H7 has not yet been reported. To determine the role of PPK1 in the pathogenesis of EHEC O157:H7, we successfully constructed a Δ*ppk1* (Figure 1B) and a complemented strain *Cppk1* (Figure 1C) to test the biological functions of PPK1 via *in vitro* and *in vivo* models.

Evaluation of the growth curve is an important but indirect method to compare the genomic expression of each strain, as the expression of many genes are affected by growth rate (Dong and Schellhorn, 2009). Our results showed that deletion of *ppk1* had little effect on the growth rate or morphology of EHEC in nutritive medium (LB) (Figure 2A). The Δ*ppk1* entered the exponential phase and stationary phase (SP) at the same time as the WT and *Cppk1* strains. However, the *ppk1* deletion mutant had reduced polyP levels as compared to the WT and *Cppk1* strains (Figure 2B). These results confirmed that at the genetic and protein level, we successfully constructed the Δ*ppk1* and *Cppk1* strains.

According to previous studies, SP is a critical period for bacteria to resist external stress and invade the host during which the function of the bacteria change significantly (Siegele and Kolter, 1992). For *E. coli*, SP is an important stage for the expression of pathogenic invasion-associated virulence genes (Wang and Kim, 2000; Peng et al., 2012). We next verified the role of PPK1 in the survival and pathogenesis of EHEC O157:H7 during SP. EHEC O157:H7 is a fecal-mouth transmitted intestinal pathogen that enters the human stomach from the external environment, passes through the intestine, and finally colonizes the colon to cause diarrhea and hemorrhagic colitis (HC), even hemolytic uremic syndrome (HUS). The premise that bacteria, such as EHEC O157:H7, causes disease was based on the thought that they survive all types of environmental stress, such as high environmental temperatures and low pH values found in the human stomach (Rao and Kornberg, 1996; Ault-Riche et al., 1998; Rao et al., 1998; Kim et al., 2002). The ability to quickly adapt to changing environments is critical for bacteria to successfully transmit and infect hosts (Dong and Schellhorn, 2009). Studies have shown that polyP is involved in the regulation of many microbial stress responses (Magnusson et al., 2005; Rao et al., 2009; Dalebroux and Swanson, 2012; Varas et al., 2017). To determine the effect of *ppk1* deletion on the bacterial stress response of EHEC O157:H7, WT, Δ*ppk1*, and *Cppk1* strains were exposed to 55°C and pH 2.0 during SP. The survival rates were compared at corresponding time points. Our results showed that the survival rates of the Δ*ppk1* strain were significantly lower than that of WT and *Cppk1* (Figures 2C,D), which indicated that PPK1 played a key role in stress response during the infection process of EHEC O157:H7.

In certain external environmental stresses, some genes of *E. coli* are induced during SP (Lange and Hengge-Aronis, 1991; Siegele and Kolter, 1992; Hengge-Aronis, 1993), such as *adAXW*, *gadB*, and *yhbO*, but are not induced during the exponential growth phase. A previous study reported that polyP was associated with the expression of RpoS, which plays a central role in SP adaptations (Abramov et al., 2007; Hoac et al., 2013). RpoS is a conserved stress regulator of virulence and pressure adaptation during SP of *E. coli*, *Salmonella typhimurium*, *Shigella*, *Yersinia colitis*, *Vibrio cholerae*, and *Borrelia burgdorferi* (Tzeng and Kornberg, 1998; Hengge-Aronis, 2002; Dong and Schellhorn, 2009). Our results show that loss of *ppk1* decreased the ability of *E. coli* to endure the stress conditions. Furthermore, real-time PCR showed decreased levels of *rpoS* in the Δ*ppk1* as compared to the WT and *Cppk1* strains (Figure 5A). This suggested that RpoS might be the stress regulator of EHEC O157:H7 and its expression was induced by polyP.

When *E. coli* is under environmental stress, RpoS initiates a complex regulatory network, inducing and regulating the expression of nearly 600 genes (Dong and Schellhorn, 2009). The expression of RpoS-regulated genes was shown to differ according to the environment. For example, the genes *gadAXW*, *gadB*, and *gadE* were found important for acid resistance (Ma et al., 2003), and *yhbO* for heat and UV resistance (Weber et al., 2005; Abdallah et al., 2007). The mechanism underlying the influence of polyP on RpoS in EHEC O157:H7 is a current focus of our research. The (p) ppGpp pathway is known to regulate other important cellular and regulatory processes that allow bacteria to adapt to a wide range of environmental conditions, including nutritional or stressful conditions (Magnusson et al., 2005; Dalebroux and Swanson, 2012). Proteomic studies conducted with *E. coli* K12 strains have identified 151 significantly differentially expressed proteins, among which RelA, SpoT, and ArcA protein levels, which influence the metabolic pathways of (p)ppGpp, were significantly decreased in the Δ*ppk1* (Varas et al., 2017). Therefore, the relationships among PPK1/polyP, RpoS and (p)ppGpp are our interest.

PPK1 was shown to be essential not only for the synthesis of polyP and metabolism of ATP, but also for adhesion/invasion and virulence factor expression in bacterial pathogens, such as *E. coli* K1 (RS218) (Peng et al., 2012), *Campylobacter jejuni* (Candon et al., 2007), *Burkholderia pseudomallei* (Srisanga et al., 2019), and *Mycobacterium tuberculosis* (Tiwari et al., 2019). The pathogenesis of EHEC O157:H7 is mainly reflected in the adhesion and effacing lesions (A/E lesions) as well as the secretion of Shiga-like toxin (Stx) (Nguyen and Sperandio, 2012).

Adhesion and effacing lesions were characterized by EHEC O157:H7 adhesion to host epithelium and then induction of extensive F-actin cytoskeletal rearrangements within cells, formation of pedestal-like structures underneath the bacteria and loss of microvillus structure (Caprioli et al., 2005). Successful adhesion and colonization of EHEC O157:H7 in the large intestine is dependent upon the type TTSS (Pacheco et al., 2018; Liu et al., 2020) by which Tir was the first translocated effector protein to the epithelial cells. Tir is inserted into the membrane of cells, where it then binds to the bacterial adhesion receptor intimin (encoded by *eae*) to mediate the adhesion between bacteria and host cells (Yi et al., 2010). Interactions between intimin and Tir were also required for recruitment and rearrangement of actin and other cytoskeletal proteins underneath adherent bacteria, which results in characteristic actin-rich “pedestals” through triggering host signal events (Ritchie et al., 2003; Golan et al., 2011).

To gain insight into the effect of *ppk1* deficiency in EHEC O157:H7 on the interaction with host cells, we elucidated whether the Δ*ppk1* mutant possessed a defect in adhesion and A/E lesion formation into host cells. Our results showed that the deletion of the *ppk1* gene reduced the adhesion as compared with WT (4.09‰ vs. 26.02‰, respectively) (Figures 3A,B). Moreover, the F-actin filaments of cells infected with the WT or *Cppk1* strains were significantly reduced and aggregated to the edge, showing a typical rearrangement phenomenon, whereas the Δ*ppk1* strain was weaker in actin filament rearrangement (Figure 3C). Accordingly, real-time PCR was carried out to test expression levels of the intimin gene *eae* in the three strains. The *ppk1* deletion significantly reduced the relative copy number of *eae* (0.32), as compared with WT (1.00) and *Cppk1* (1.06) (Figure 5), which was consistent with these results at the cellular level. In animal models, deletions of *eae* (the intimin locus) and mutations that render the TTSS inactive markedly reduced the pathogen’s capacity to colonize the intestine (Ritchie et al., 2003), which was similar to our results above.

When colonizing the human intestine, EHEC O157:H7 forms A/E lesions on the colonic epithelial cells. The genes required for A/E lesions were encoded within the chromosomal pathogenicity island known as the locus for enterocyte effacement (LEE) (Elliott et al., 1998; Muller et al., 2009; Nguyen and Sperandio, 2012). The secreted effector proteins by the type III secretion system within LEE were directly injected into the cells through a “molecular injector”. One of the important secreted proteins injected into the host was the translocated intimin receptor (Tir) (Nguyen and Sperandio, 2012). Once released into the host cytoplasm, Tir localizes to the host cytoplasmic membrane and binds to the LEE-encoded surface protein intimin (encoded by *eae*) to intimately attach the bacteria to the cell (Kenny et al., 1997; Deibel et al., 1998; Nguyen and Sperandio, 2012). Together, with other effector proteins secreted into the cell, F-actin was aggregated, leading to rearrangement of the cytoskeleton of the colonic epithelial cell (Campellone et al., 2004b; Weiss et al., 2009). Actin rearrangement was followed by the appearance of characteristic pathological manifestation of A/E lesions, including intestinal epithelial cytoskeletal (F-actin) rearrangement, destruction of tight junctions, intestinal microvilli actin dimerization, microvilli loss (Rao and Kornberg, 1996), and a pedestal-like structure formation, which cup the individual bacteria (Nataro and Kaper, 1998; Nguyen and Sperandio, 2012). Our results indicated that *ppk1* deletion mutant downregulated the *eae* gene and then reduced the expression of intimin. As a result, Tir could not bind to sufficient intimin, leading to the decrease in adhesion of Δ*ppk1* to LoVo cells. Adhesion is an important step of A/E lesions, which directly affects the rearrangement of F-actin and formation of the pedestal. Therefore, the rearrangement of F-actin in Δ*ppk1* was not obvious, resulting in weak A/E lesions. Thus, PPK1 played an important role in the adhesion and formation of A/E lesions which was verified in other bacteria (Candon et al., 2007; Peng et al., 2012; Tiwari et al., 2019).

Aside from A/E lesions, another main pathogenic factor of EHEC O157:H7 causing severe diarrhea and HUS is the secretion of Shiga-like toxins (Stx1 and Stx2) (Leotta et al., 2008). The Stx molecule consists of one A subunit and five B subunits through covalent bonding via an AB5 structure, and its toxic effect on host cells has already been widely recognized (Laing et al., 2012; Page and Liles, 2013; Perera et al., 2015). Briefly, after adhesion and colonization in the colon, STEC, such as EHEC O157:H7, induced the delivery of Stx, which passes through the intestinal mucosa. Then toxins entered the bloodstream and traveled to target organs such as kidneys with toxin receptor Gb3 on the cell surface. Once bounded to Gb3, Stxs were internalized and delivered from early endosome to the trans-Golgi network, through the Golgi apparatus, to the endoplasmic reticulum (ER), leading to inhibition of protein synthesis, ER stress, ribotoxic stress, pro-inflammatory responses, and autophagy in host cells (Leyva-Illades et al., 2012; Lee et al., 2016, 2021). Therefore, Stx causes severe inflammation and extensive histological damage, resulting in bloody dysentery or bloody diarrhea and acute renal failure. In addition, Stx also causes programmed cell death or apoptosis (Jones et al., 2000; Suzuki et al., 2000).

Moreover, Stx maybe a virulence factor to affect intestinal tissue damage alone (Lee et al., 2021). Gigliucci et al. (2018) detected *stx2a* (encoding Stx2a) and *eae* (encoding intimin) in the feces of STEC-infected patients, which suggested that Stx not only works by migrating to the lamina propria but can also act in the intestinal lumen. Robinson et al. (2006) found that Stx2 was involved in increasing the colonization capacity of EHEC by increasing the expression of nucleolin in HEp-2 cells, which implied that Stxs affected the EHEC colonization of the intestine (Lee et al., 2021).

To determine the influence of the deletion of the *ppk1* gene on the Stx virulence of EHEC O157:H7, real-time reverse-transcription PCR was performed. Our results showed that the *ppk1* deletion significantly reduced the relative copy number of *stx1A*, *stx1B*, *stx2A*, and *stx2B* as compared to the WT and *Cppk1* strains (Figure 5). This suggested that *ppk1* affected the virulence of EHEC O157:H7 at a molecular level. Moreover, to identify the effect of *ppk1* deletion on the bacterial invasion of cells, an invasion assay was performed via TEM and gentamicin protection experiments. Our results demonstrated that the invasion rate of Δ*ppk1* was reduced to 0.39‰ as compared with WT (0.56‰) and *Cppk1* (0.62‰) (Figures 3B,D).

Enterohemorrhagic *Escherichia coli* invasions is sometimes associated with recurrent infection and persistent diarrhea. It attained some degree of coexistence with the infected cell and survived in the intracellular milieu for a long time. That strategy seemed appropriate to assure the persistence of the micro-organism in the animal reservoir, as well as to sustain its continuous shedding to the environment (Cordeiro et al., 2013). On the other hand, host cell cytoskeletal rearrangement and phosphorylation-mediated signal transduction are also involved in cell invasion by pathogens, which leads to the intestinal injury (Cossart and Sansonetti, 2004). Based on the adhesion, A/E lesions and invasion results, we concluded that the Δ*ppk1* was less adherent and invasive in LoVo cells. We further confirmed the effect of *ppk1* deletion on intestinal injury through animal experiments.

Three commonly used species (pigs, rabbits, and mice) as animal models have been developed to facilitate study of EHEC pathogenesis *in vivo*. The piglet intestine is permissive for EHEC replication and shows typical signs of central nervous system (CNS) as human beings (Ritchie, 2014). But their breeding and maintenance require considerable veterinary skill, space, and financial support (Mohawk and O’Brien, 2011). Rabbits have been used both to study the toxic effects of Stx and the intestinal biology of the organism. Despite being sensitive to the intestinal manifestations of EHEC infection, suckling rabbits do not develop HUS or any other evidence of renal failure. Mouse is another small animal model systems are preferable for general use. Mice are naturally resistant to colonization by EHEC and fail to develop signs of intestinal disease following oral infection. Thus, we tried to use mitomycin C (MMC) (an alkylating agent regarded as an important chemotherapeutic drug and is widely used in the treatment of several malignant human tumors) as an agent to induce toxin expression and form animal model of EHEC O157:H7 infection (Fujii et al., 1994; Shimizu et al., 2003).

Our experimental results showed that the diarrhea symptoms of WT and *Cppk1* mice were obvious, but the diarrhea symptoms of Δ*ppk1* were not, which could be seen on fecal formation (Figure 4A). Moreover, colonic HE staining and histologic damage scores showed that Δ*ppk1* was less destructive to the colon, which was also an evidence of mild symptoms of enteritis, as shown in Figure 4B.

All these results indicated that PPK1 was a key factor in the pathogenesis of EHEC O157:H7. The *ppk1*-deficient strain exhibited defects in adhesion and invasion in human colonic epithelial cells, which was consistent with the results of others (Dong and Schellhorn, 2009; Peng et al., 2012; Srisanga et al., 2019).

In summary, we demonstrated that *ppk1* was involved in the pathogenesis of EHEC O157:H7. PPK1 improved the stress response by regulating the expression of the stable phase regulatory gene *rpoS*, promoted A/E lesions, and increased the expression of virulence genes *stx1 and stx2*, which enhanced the pathogenicity of the bacteria. However, further research regarding the mechanism of PPK1-mediated regulation will improve our understanding of the pathogenesis and aid in the prevention of EHEC O157:H7 infection. PPK1 is not present in humans, and as such, the unique ATP-binding pocket in its structure is an important target for the development of PPK1 inhibitors, especially for new antibiotics. This study identified PPK1 as a potential target for the development of novel treatments for EHEC O157:H7 infection.

## Data Availability Statement

The original contributions presented in the study are included in the article/supplementary material, further inquiries can be directed to the corresponding author/s.

## Ethics Statement

The animal study was reviewed and approved by Southern Medical University Experimental Animal Ethics Committee.

## Author Contributions

YD and XW did the conception and design of the work and drafted the article. YD, YH and KY collected the data. ZH, BZ, and WZ did the data analysis and interpretation. CW did the critical revision of the article. WZ and CW did the final approval of the version to be published. All authors contributed to the article and approved the submitted version.

## Conflict of Interest

The authors declare that the research was conducted in the absence of any commercial or financial relationships that could be construed as a potential conflict of interest.

## Publisher’s Note

All claims expressed in this article are solely those of the authors and do not necessarily represent those of their affiliated organizations, or those of the publisher, the editors and the reviewers. Any product that may be evaluated in this article, or claim that may be made by its manufacturer, is not guaranteed or endorsed by the publisher.
